# Cross-Sectional Associations of Depressive Symptom Severity and Functioning with Health Service Use by Older People in Low-and-Middle Income Countries

**DOI:** 10.3390/ijerph120403774

**Published:** 2015-04-02

**Authors:** Conal D. Twomey, Martin Prince, Alarcos Cieza, David S. Baldwin, A. Matthew Prina

**Affiliations:** 1 Faculty of Social and Human Sciences, School of Psychology, University of Southampton, Southampton SO17 1BJ, UK; E-Mail: a.cieza@soton.ac.uk; 2 Centre for Global Mental Health, Health Service and Population Research Department, Institute of Psychiatry, Psychology and Neuroscience, King’s College London, London SE5 8AF, UK; E-Mails: martin.prince@kcl.ac.uk (M.P.); matthew.prina@kcl.ac.uk (A.M.P.); 3 Research Unit for Biopsychosocial Health, Department of Medical Informatics, Biometry and Epidemiology—IBE, Ludwig-Maximilians-University (LMU), Munich 81377, Germany; 4 Swiss Paraplegic Research (SPF), Nottwil 6207, Switzerland; 5 Faculty of Medicine, University of Southampton, Southampton SO17 1BJ, UK; E-Mail: d.s.baldwin@soton.ac.uk

**Keywords:** depression, functioning, health service use, low-and-middle income countries, cross-sectional

## Abstract

*Background*: Comprehensive understanding of the determinants of health service use (HSU) by older people with depression is essential for health service planning for an ageing global population. This study aimed to determine the extent to which depressive symptom severity and functioning are associated with HSU by older people with depression in low and middle income countries (LMICs). *Methods:* A cross-sectional analysis of the 10/66 Dementia Research Group population-based surveys dataset. Participants (*n* = 4590) were those aged 65 or older, in the clinical range for depressive symptoms (defined as scoring four or more on the EURO-D), living in 13 urban and/or rural catchment areas in nine LMICs. Associations were calculated using Poisson regression and random-effects meta-analysis. *Results*: After adjustment for confounding variables, (EURO-D) depressive symptom severity was significantly associated with “any community HSU” (Pooled Prevalence Ratios = 1.02; 95% CI = 1.01–1.03) but not hospital admission. Conversely, after adjustment, (WHODAS-II) functioning was significantly associated with hospital admission (Pooled PR = 1.14; 95% CI = 1.02–1.26) but not “any community HSU”. *Conclusions*: Depressive symptom severity does not explain a large proportion of the variance in HSU by older people with depression in LMICs. The association of functioning with this HSU is worthy of further investigation. In LMICs, variables related to accessibility may be more important correlates of HSU than variables directly related to health problems.

## 1. Introduction

Older people- here defined as people aged 65 years or older- consume substantially more health service resources than younger people. *Per capita*, health service use (HSU) by older people is 3–5 times higher than in younger people [1]. In England, older people account for 45% of hospital admissions and nearly two thirds of hospital bed-days [2,3]. Although better health patterns in old age should be taken into account [4], the ongoing demographic shift towards an older global population will increasingly place greater demands on health services [5]. Therefore, a comprehensive understanding of the variables associated with HSU by older people is essential for effective health service planning over the long term.

One of the most common and burdensome health problems experienced by older people is depression. Up to 25% of older people suffer from major depression, dysthymic disorder, or subthreshold depression [6]. Due to the ageing population, it has been projected that between the year 2000 and 2050, the number of older people with depression will increase by 117% [7]. Depression in older people is highly burdensome, and associated with poor quality of life, disability, and increased likelihood of physical health comorbidities [8]. Moreover, depression has various biological effects (e.g., alteration of cardiac functioning, inflammation, impairments in cell-mediated immunity) and is associated with risk factors for chronic disease (e.g., smoking, poor diet, hypertension) [9].

Given that the commonality and burden of depression in older people has important implications for health service planning, there has been a relative lack of research investigating the association of depressive symptom severity with HSU in older people. Moreover, findings from the few existing general population studies examining this association are somewhat inconsistent. Across HSU outcomes (e.g., community-based HSU, hospital admissions), most studies provide evidence for this association [10,11,12] but some do not [13,14,15]. Although a recent meta-analysis of seven studies found an association of depressive symptom severity with hospital admissions, the magnitude of this association was small (RR = 1.36) [11].

Previous research suggests that depressive symptom severity may not explain a large amount of the variance in HSU by older people with depression, and there is a need to determine the extent to which other variables may explain the remaining variance. One variable worthy of investigation in this regard is “functioning”, an encompassing term relating to body functions, body structures and activities and participation [16]. Functioning is relevant because it reflects the burden of all health problems (which tends to increase with age), meaning that it is potentially associated with utilisation of a diverse range of health services. Various studies have demonstrated positive associations of functioning impairment (operationalised in differing ways) with HSU by clinical and general populations [17,18,19] and depressive symptom severity in older people [20,21,22]. However, although one recent study included analysis of the association of physical impairments with HSU by older people [23], few studies have so far directly examined the association of functioning with HSU by older people with depression [11].

The majority of previous research has taken place in high income countries and there is a need to increase knowledge of the variables associated with HSU by older people with depression in low and middle income countries (LMICs), where momentum for health care reforms aimed at achieving universal health care coverage is growing [24]. Available findings are mostly based on data from the general population (e.g., community-dwelling older people) rather than participants with probable depression. A sole focus on participants with probable depression would facilitate the examination of HSU patterns specifically related to depression, adding precision to subsequent findings.

The present study encompasses a cross-sectional analysis of the 10/66 Dementia Research Group population-based surveys dataset [25] and addresses the three research gaps mentioned above. Our main aim was to determine the extent to which depressive symptom severity and functioning are associated with HSU by older people in LMICs. To explore further the (unclear) impacts of functioning on this HSU, we also aimed to determine if specific functioning difficulties are more related to this HSU than others.

## 2. Methods

### 2.1. Participants and Procedures

Prince *et al.* [25] have provided the full protocol of the 10/66 Dementia Research Group population-based surveys. Undertaken between 2003 and 2005, the surveys provide data from a sample of people aged 65 years and over living in 13 urban and/or rural catchment areas in nine LMICs (China, Cuba, Dominican Republic, India, Mexico, Nigeria, Peru, Puerto Rico, Venezuela) [25]. Urban sites were of high density and low socioeconomic status, and rural sites were of low density, representing a traditional agrarian lifestyle. Selected sites had mapped boundaries, and systematic door-knocking identified eligible participants (*i.e.*, those aged 65 and over). Surveys consisted of questionnaires, structured clinical interviews, an informant interview, and a physical examination. Data collected pertained to demographics, physical health, mental health (e.g., depression, dementia), chronic disease risk factors, functioning impairment, HSU, care arrangements and caregiver strain. All studies were approved locally and also by the Institute of Psychiatry, King’s College London. Data from over 17,000 participants are provided through the surveys. However, in the present study, only participants with elevated symptoms of depression (*n* = 4950) were included: participants had to score 4 or more on the EURO-D scale [26], described below (Section 2.2.2).

### 2.2. Measures

#### 2.2.1. Sociodemographics

Age, gender, education (stratified by education levels; e.g., “no education”, “primary education”, “secondary education”), marital status, household assets owned, pension coverage, private health insurance coverage, number of physical comorbidities (grouped as “none”, “one to two” or “three and above”), “any diagnosis of dementia”, and “previous ICD-10 depressive episode” were used to assess the sociodemographic background of participants.

#### 2.2.2. Depression

Depressive symptoms were measured using the 12-item EURO-D scale [26], derived from the Geriatric Mental State examination [27]. The EURO-D addresses the symptoms of depressed mood, pessimism, suicidality, guilt, sleep, interest, irritability, appetite, fatigue, concentration, enjoyment, and tearfulness. Individual items are scored according to absence (0) or presence (1) of these symptoms and the total score ranges from 0–12. The EURO-D has been validated cross-culturally, in both high income countries and LMICs, with a score of four or more representing the presence of probable depression [26,28,29].

#### 2.2.3. Functioning

Functioning was measured using the 12-item World Health Organization Disability Assessment Schedule II (WHODAS-II) which addresses difficulties with: standing, taking care of household responsibilities, learning a new task, joining in community activities, emotionality, concentration, walking a long distance, washing, dressing and undressing, relating to unknown people, maintaining friendships and the performing of day-to-day work/school [30]. Individual items are scored on a five-point scale (1 = none; 2 = mild; 3 = moderate; 4 = severe, 5 = extreme/cannot do) and the total score is expressed as a percentage representing the degree of functional impairment experienced. This widely-used, reliable, and valid measure has been psychometrically evaluated in the 10/66 Dementia Research Group study population, generating a one-factor solution in most sites [31].

#### 2.2.4. HSU

HSU was measured using the Client Services Receipt Inventory [32], in a version adapted for use in LMICs [33]. Participants were asked to recall whether or not they had a contact with any community health services (*i.e.*, primary care, hospital doctor/outpatient, private doctor, other community services, traditional healer) or if they had been admitted to hospital, in the previous three months.

### 2.3. Selection of Potential Confounding Variables

Based on previous research showing their associations with HSU by older people in LMICs [23], potential confounding variables (*i.e.*, age, gender, education level, physical comorbidities, private insurance coverage, pension coverage and “any dementia diagnosis”) were included in initial model-building for adjustment purposes. However, to ensure statistical power and precision, the decision was made to remove variables from the model. Thus, after sensitivity analysis showed that pension coverage and “any dementia diagnosis” did not explain meaningful levels of additional variance in outcomes, these variables were removed. It was not possible to include private insurance coverage because there were too few cases (or analysable participants) available to estimate parameters at various sites.

### 2.4. Statistical Analysis

All analyses were conducted using STATA 13 StataCorp LP, College Station, TX, USA), and release 3.3 of the 10/66 dataset. For each site, descriptive statistics summarised sociodemographics and previous HSU (which was also standardised for age, gender, and education, using the whole sample from all sites as the external standard population). Pooled, across-site, HSU descriptive data were also calculated through random-effects meta-analysis [34]. For each site, the associations of depressive symptom severity, functioning and functioning items with HSU were determined using unadjusted and adjusted (for potential confounding variables) prevalence ratios (and 95% confidence intervals) modelled through Poisson regression analysis which controlled for household clustering. References in comparisons were a one-point increase on the EURO-D or a 10-point increase on the WHODAS-II, where appropriate. Poisson regression was chosen as it is appropriate for analysis of dichotomous count data and facilitates the calculation of prevalence ratios which are relatively easy to interpret. Pooled, across-site, unadjusted and adjusted prevalence ratios were then calculated through random-effects meta-analysis. Using random-effects meta-analysis allowed heterogeneity at site level to be accounted for in overall estimates. For meta-analyses, Higgins I^2^ [35] was used to determine the percentage of variance in study estimates attributable to heterogeneity.

## 3. Results

### 3.1. Sociodemographic and Clinical Characteristics of Participants

Table 1 summarises participant sociodemographic and clinical information for each study site. In total, 4590 participants scored 4 or more on the EURO-D scale [26] and were included in analyses. The smallest sample size was in “China (urban)” (*n* = 15) and the largest sample size was in the Dominican Republic (*n* = 757). In terms of age, 29.3% of participants were aged 65 to 69, 26.6% were aged 70–74, 20.5% were aged 75–79, and 23.3% were aged 80 or over. Most participants were female (71.5%). The majority of participants had received some education (73.5%) but relatively few had completed secondary education or above (17.6%). In terms of marital status, the majority were either married or widowed (81.2%). 65.9% of participants had five or more household assets (*i.e.*, car, television, refrigerator, telephone, mains water, mains electricity, plumbed toilet) and 45.7% had a pension, but 69.8% had no private insurance. Most participants had at least one physical comorbidity (70.6%). A minority had a dementia diagnosis (13.4%). Although all participants were in the clinical range for depressive symptoms (as per inclusion criteria), only a minority had a previous ICD-10 depressive episode (20.3%).

### 3.2. Previous HSU

Table 2 summarises previous HSU by participants. Across sites, the prevalence for contact with any community health service in the previous three months was 57.8% (95% CI = 46.6–69.0). After direct standardisation for age, gender and education (using the whole sample from all sites as the external standard population), the prevalence of HSU was 50.2% (95% CI = 36.6–63.7). Across sites, the crude prevalence for hospital admission in the previous three months was 3.6% (95% CI = 2.5–4.6) and the standardised prevalence was 3.4% (95% CI = 1.9–4.7).

### 3.3. Associations of Depressive Symptom Severity and Functioning with HSU

Table 3 reports the associations of depressive symptom severity (EURO-D) [26] and functioning (WHODAS-II) [30] with HSU. The site “China (rural)” was excluded from these analyses because there were too few cases available to estimate parameters. For the “any community HSU” outcome, significant associations were found for both depressive symptom severity (Pooled PR = 1.02; 95% CI = 1.01–1.03; I^2^ = 14.0%) and functioning (Pooled PR = 1.02; 95% CI = 1.01–1.04; I^2^ = 71.2%). After adjustment for age, gender, education, and physical comorbidities, the association remained significant for depressive symptom severity (Pooled PR = 1.02; 95% CI = 1.01–1.03; I^2^ = 20.8%) but not functioning (Pooled PR = 1.01; 95% CI = 1.00–1.03; I^2^ = 44.7%).

For the hospital admission outcome, no significant association was found for depressive symptom severity (Pooled PR = 1.11; 95% CI = 0.96–1.25; I^2^ = 70.2%) but a significant association was found for functioning (Pooled PR = 1.18; 95% CI = 1.07–1.29; I^2^ = 64.9%). After adjustment for age, gender, education, and physical comorbidities, similar results were found (Table 3).

To assess the validity of using the (EURO-D) [26] for measuring the association of depressive symptom severity with HSU, sensitivity analyses of this association were performed. Here depressive symptom severity was measured through “previous ICD-10 depressive episode”. Similar to the previous analyses, depressive symptom severity (“previous ICD-10 depressive episode”) was significantly associated with “any community HSU” (Pooled PR = 1.05; 95% CI = 1.01–1.10; I^2^ = 44.5%) but not hospital admissions (Pooled PR = 1.37; 95% CI = 0.73–2.02; I^2^ = 0%).

### 3.4. Associations of Specific Functioning Difficulties with “Any Community HSU”

Table 4 reports mutually-adjusted associations of the 12 functioning items (WHODAS-II) [30] with the “any community HSU” outcome. The site “China (rural)” and the hospital admission outcome were excluded from analyses because there were too few cases available to estimate parameters. Only one item, “emotionally affected by health problems”, was significantly associated with increased “any community HSU” (Pooled PR = 1.06; 95% CI = 1.03–1.09; I^2^ = 31.7%). After adjustment for age, gender, education, and physical comorbidities, this association remained significant (Pooled PR = 1.04; 95% CI = 1.01–1.07; I^2^ = 22.4%). The item “difficulty with washing whole body” was significantly associated with decreased “any community HSU”, to a similar level before and after adjustment (Pooled PR = 0.93; 95% CI = 0.88–0.98; I^2^ = 31.7%).

**Table 1:** Sociodemographic and clinical characteristics of participants.

Variable	*n* (%)													
	All	China (Urban)	China (Rural)	Cuba	Dominic.Republic	India (Urban)	India (Rural)	Mexico (Urban)	Mexico (Rural)	Nigeria	Peru (Urban)	Peru (Rural)	Puerto Rico	Vene-zuela
***n***	4590	44	15	682	757	390	422	312	259	270	390	144	331	574
**Age (MV)**	5	0	0	1	0	2	0	1	0	0	0	0	0	1
65–69	1347	16	3	182	189	146	140	64	81	82	91	42	89	222
	(29.3)	(36.3)	(20.0)	(26.6)	(24.9)	(37.4)	(33.1)	(20.5)	(31.2)	(30.3)	(23.3)	(29.1)	(26.8)	(38.6)
70–74	1224	11	8	151	173	130	152	106	65	76	99	40	69	144
	(26.6)	(25.0)	(53.3)	(22.1)	(22.8)	(33.3)	(36.0)	(33.9)	(25.1)	(28.1)	(25.3)	(27.7)	(20.8)	(25
75–79	941	7	3	164	161	61	75	69	57	35	99	29	81	100
	(20.5)	(15.9)	(20.0)	(24.0)	(21.2)	(15.6)	(17.7)	(22.1)	(22)	(12.9)	(25.3)	(20.1)	(24.4)	(17.4)
80–84	576	3	1	105	115	28	38	43	32	39	55	21	47	49
	(12.5)	(6.8)	(6.6)	(15.4)	(15.1)	(7.1)	(9.0)	(13.7)	(12.3)	(14.4)	(14.1)	(14.5)	(14.2)	(8.5)
85–89	345	7	0	55	76	15	14	24	18	22	34	8	32	40
	(7.5)	(15.9)		(8.0)	(10.0)	(3.8)	(3.3)	(7.6)	(6.9)	(8.1)	(8.7)	(5.5)	(9.6)	(6.9)
90+	152	0	0	24	43	8	3	5	6	16	12	4	13	18
	(3.3)			(3.5)	(5.6)	(2.0)	(0.7)	(1.6)	(2.3)	(5.9)	(3.0)	(2.7)	(3.9)	(3.1)
**Gender (MV)**	7	0	0	0	0	0	0	0	0	0	0	0	0	0
Female	3282	27	6	535	573	261	232	235	196	165	282	92	262	416
	(71.5)	(61.3)	(40.0)	(78.4)	(75.6)	(66.9)	(54.9)	(75.3)	(75.6)	(61.1)	(72.3)	(63.8)	(79.1)	(72.4)
**Education (MV)**	20	0	0	0	0	0	0	0	0	18	0	0	2	0
No education	1217	10	6	28	186	220	294	91	93	178	12	33	17	49
	(26.5)	(22.7)	(40.0)	(4.1)	(24.5)	(56.4)	(69.6)	(29.1)	(35.9)	(65.9)	(3.0)	(22.9)	(5.1)	(8.5)
Some	1351	10	5	182	398	83	75	130	133	32	42	40	67	154
	(29.5)	(22.7)	(33.3)	(26.6)	(52.5)	(21.2)	(17.7)	(41.6)	(51.3)	(11.8)	(10.7)	(27.7)	(20.2)	(26.8)
Completed primary	1155	14	4	247	108	60	40	59	25	32	157	56	82	271
	(25.2)	(31.8)	(66.6)	(36.2)	(14.2)	(15.3)	(9.4)	(18.9)	(9.6)	(11.8)	(40.2)	(38.8)	(24.7)	(47.2)
Completed secondary	540	6	0	136	43	21	12	20	5	6	122	7	110	52
	(11.8)	(13.6)		(19.9)	(5.6)	(5.3)	(2.8)	(6.4)	(1.9)	(2.2)	(31.2)	(4.8)	(33.2)	(9.0)
Completed tertiary	270	4	0	88	14	6	1	12	3	4	55	5	53	25
	(5.8)	(9.0)		(12.9)	(1.8)	(1.5)	(0.2)	(3.8)	(1.1)	(1.4)	(14.1)	(3.4)	(16)	(4.3)
Unknown	37	0	0	1	8	0	0	0	0	0	2	3	0	23
	(0.8)			(0.1)	(1.0)						(0.5)	(2.0)		(4.0)
**Marital Status (MV)**	47	0	0	1	4	0	0	0	0	18	1	0	2	21
Never married	265	0	0	69	37	8	2	16	10	9	42	10	15	47
				(10.1)	(4.8)	(2.0)	(0.4)	(5.1)	(3.86)	(3.30)	(10.7)	(6.9)	(4.5)	(8.1)
	(5.7)													
Married/Cohabiting	1882	30	4	232	182	153	199	140	121	162	196	85	158	220
	(41.0)	(68.1)	(26.6)	(34.0)	(24.0)	(39.2)	(47.1)	(44.8)	(46.72)	(60.0)	(50.2)	(59.0)	(47.7)	(38.3)
Widowed	1847	14	11	257	348	206	212	127	110	81	128	47	116	190
	(40.2)	(31.8)	(73.3)	(37.6)	(45.9)	(52.8)	(50.2)	(40.7)	(42.47)	(30.0)	(32.8)	(32.6)	(35.0)	(33.1)
Divorced/Separated	549	0	0	123	186	23	9	29	18	0	23	2	40	96
	(11.9)			(18.0)	(24.5)	(5.9)	(2.1)	(9.2)	(6.9)		(5.9)	(1.3)	(12)	(16.7)
**Assets ^1^ (MV)**	219	0	0	1	2	2	0	0	0	214	0	0	0	0
0	72	0	0	2	3	3	43	0	1	4	0	2	0	14
	(1.6)			(0.3)	(0.4)	(0.8)	(10.2)		(0.4)	(1.5)		(1.4)		(2.4)
1–4	1274	3	5	72	281	265	351	36	155	43	9	50	4	560
	(27.8)	(6.8)	(33.3)	(10.5)	(37.1)	(67.9)	(83.2)	(11.5)	(59.8)	(15.9)	(2.3)	(34.7)	(1.2)	(97.6)
5+	3025	41	41	607	471	120	28	276	103	9	381	92	327	0
	(65.9)	(93.2)	(93.2)	(89.0)	(62.2)	(30.8)	(6.6)	(88.5)	(39.8)	(3.3)	(97.7)	(63.9)	(98.8)	
**Pension (MV)**	0	0	0	0	0	0	0	0	0	0	0	0	0	0
Yes	2098	38	3	541	208	23	145	228	78	1	234	100	190	309
	(45.7)	(86.4)	(20)	(79.3)	(27.5)	(5.9)	(34.4)	(73.1)	(30.1)	(0.4)	(60)	(69.4)	(57.4)	(53.8)
**Private Insurance** (MV)	63	0	0	2	1	2	0	0	0	29	2	0	2	25
None	3204	44	9	680	642	387	422	134	184	241	88	33	19	321
	(69.8)	(100)	(60.0)	(99.7)	(84.8)	(99.2)	(100)	(42.9)	(71.0)	(89.2)	(22.5)	(22.9)	(5.7)	(55.9)
**Physical Comorbidities (MV)**	27	0	0	1	0	0	0	0	0	2	1	0	4	19
1–2	1954	18	8	382	342	147	204	135	113	139	166	74	37	189
	(42.6)	(40.9)	(53.3)	(56.0)	(45.2)	(37.7)	(48.3)	(43.3)	(43.6)	(42.0)	(42.6)	(51.4)	(13.7)	(32.9)
3+	1287	19	4	122	310	28	101	92	85	131	117	23	7	248
	(28.0)	(43.2)	(26.7)	(17.9)	(40.9)	(7.2)	(23.9)	(29.5)	(32.8)	(39.6)	(30.0)	(15.9)	(2.6)	(43.2)
**Dementia (MV)**	1	0	0	0	0	0	0	0	0	0	0	0	1	0
Any diagnosis	615	10	10	98	121	43	52	45	32	42	32	19	42	69
	(13.4)	(22.7)	(66.7)	(14.4)	(16.0)	(11.0)	(12.3)	(14.4)	(12.4)	(15.6)	(8.2)	(13.2)	(12.7)	(12.0)
**Depression (MV)**	0	0	0	0	0	0	0	0	0	0	0	0	0	0
ICD-10 depressive episode	931	3	6	142	274	37	124	46	42	5	84	16	46	106
	(20.3)	(6.8)	(40.0)	(20.8)	(36.2)	(9.5)	(29.4)	(14.7)	(16.2)	(1.9)	(21.5)	(11.1)	(13.9)	(18.5)

**Note:** MV = Number of missing values. **^1^** Household assets (*i.e.*, car, television, refrigerator, telephone, mains water, mains electricity, plumbed toilet).

**Table 2:** Prevalence of previous health service utilisation.

Country	*n*	Any Community Health Service ^1^ (MV = 0)				Hospital Admission (MV = 27)			
		Crude		Standardised ^2^		Crude		Standardised ^2^	
		%	(95% CI)	%	(95% CI)	%	(95% CI)	%	(95% CI)
**China (urban)**	44	59.0	(44.9–73.3)	37.1	(34.2–39.9)	13.6	(3.4–23.8)	6.7	(2.2–11.2)
**China (rural)**	15	6.6	(0–19.3)	2.1	(0–5.6)	0	-	0	-
**Cuba**	682	53.6	(49.9–57.5)	51.4	(46.0–56.9)	2.8	(1.5–4.0)	2.4	(1.2–3.7)
**Dominican Republic**	757	52.3	(48.7–55.9)	52.8	(49.0–56.6)	4.3	(2.9–5.8)	4.4	(2.8–6.1)
**India (urban)**	390	55.9	(50.8–60.9)	50.5	(45.7–55.3)	1.2	(0.2–2.3)	0.9	(0–1.8)
**India (rural)**	422	91.0	(88.3–93.7)	60.3	(57.1–63.6)	1.9	(0.6–3.1)	0.7	(0.2–1.2)
**Mexico (urban)**	237	79.4	(74.9–83.9)	73.7	(69.9–77.6)	4.1	(1.9–6.3)	3.4	(1.5–5.3)
**Mexico (rural)**	259	68.3	(62.6–74.0)	61.7	(57.6–65.7)	2.7	(0.7–4.6)	1.4	(0.5–2.3)
**Nigeria**	270	24.4	(19.4–29.4)	23.6	(19.2–27.9)	4.1	(1.7–6.5)	2.7	(1.4–4.1)
**Peru (urban)**	223	60.0	(54.9–65.0)	49.1	(43.9–4.4)	3.8	(1.8–5.8)	7.4	(6.4–8.4)
**Peru (rural)**	144	40.2	(32.2–48.4)	37.2	(32.1–42.2)	2.1	(−2.5–4.4)	1.8	(0.01–3.5)
**Puerto Rico**	331	83.9	(80.0–87.9)	82.1	(77.9–86.2)	6.1	(3.5–8.6)	4.5	(2.3–6.8)
**Venezuela**	574	68.8	(65.0–72.6)	70.4	(65.6–75.0)	6.4	(4.6–8.7)	5.4	(3.6–7.2)
**Pooled estimate**	4590	57.8	(46.6–69.0)	50.2	(36.6–63.7)	3.6	(2.5–4.6)	3.4	(1.9–4.7)

**Notes:** **^1^** Includes contacts with primary care, private doctor, hospital doctor, traditional healer, other services, in the previous 3 months; **^2^** Standardised for age (groups), gender, and education; MV = Number of missing values.

**Table 3:** Associations of depressive symptom severity (EURO-D total score) and functioning (WHODAS-II total score) with health service utilisation.

Country	*n*	Prevalence Ratios (95% CI)							
		Any Community Health Service ^1^ (MV = 0)				Hospital Admission(MV = 27)			
		Depression Severity		Functioning ^2^		Depression Severity		Functioning ^2^	
		Crude	Adjusted ^3^	Crude	Adjusted ^3^	Crude	Adjusted ^3^	Crude	Adjusted ^3^
**China (urban)**	44	0.92	0.92	0.97	0.99	1.35	1.30	1.52	1.21
		(0.77–1.09)	(0.78–1.08)	(0.88–1.07)	(0.88–1.11)	(0.94–1.95)	(0.81–2.11)	(0.92–1.67)	(0.82–1.78)
**Cuba**	682	1.03	1.04	0.96	0.96	0.93	0.93	0.89	0.83
		(0.99–1.07)	(1.00–1.08)	(0.93–0.99)	(0.93–1.01)	(0.73–1.19)	(0.73–1.19)	(0.75–1.07)	(0.68–1.02)
**Dominican Republic**	757	1.02	1.02	1.03	1.02	1.21	1.18	1.27	1.28
		(0.98–1.06)	(0.98–1.06)	(1.00–1.05)	(0.98–1.04)	(1.04–1.40)	(1.01–1.37)	(1.14–1.42)	(1.11–1.47)
**India (urban)**	390	1.03	1.05	1.08	1.03	1.54	1.87	1.39	1.12
		(0.98–1.09)	(1.00–1.10)	(1.03–1.11)	(0.98–1.07)	(1.29–1.84)	(1.54–2.27)	(1.06–1.81)	(0.84–1.48)
**India (rural)**	422	1.01	1.02	0.99	0.99	1.08	1.01	1.19	1.12
		(0.99–1.03)	(1.00–1.04)	(0.97–1.01)	(0.97–1.01)	(0.82–1.43)	(0.77–1.33)	(0.81–1.76)	(0.76–1.63)
**Mexico (urban)**	237	1.01	1.01	1.00	1.00	0.91	0.93	1.22	1.26
		(0.98–1.04)	(0.98–1.04)	(0.97–1.02)	(0.97–1.03)	(0.71–1.16)	(0.71–1.22)	(0.99–1.50)	(1.04–1.53)
**Mexico (rural)**	259	0.99	1.00	1.00	1.02	1.28	1.36	1.29	1.32
		(0.94–1.05)	(0.94–1.05)	(0.97–1.04)	(0.99–1.05)	(0.95–1.74)	(0.95–1.95)	(1.09–1.52)	(1.09–1.61)
**Nigeria**	270	0.96	0.95	1.07	1.11	0.73	0.76	0.96	0.92
		(0.87–1.05)	(0.87–1.04)	(0.98–1.17)	(1.02–1.21)	(0.57–0.94)	(0.58–0.98)	(0.77–1.17)	(0.73–1.17)
**Peru (urban)**	223	1.06	1.06	1.05	1.05	1.42	1.44	1.27	1.39
		(1.01–1.10)	(1.01–1.10)	(1.01–1.09)	(1.02–1.09)	(1.11–1.82)	(1.06–1.94)	(1.09–1.49)	(1.18–1.64)
**Peru (rural)**	144	1.19	1.19	1.12	1.05	0.89	0.82	0.75	0.73
		(1.03–1.36)	(1.04–1.36)	(1.03–1.23)	(0.95–1.14)	(0.45–1.76)	(0.43–1.52)	(0.47–1.20)	(0.42–1.28)
**Puerto Rico**	331	1.03	1.02	1.02	1.01	1.01	0.98	1.30	1.24
		(1.00–1.05)	(1.00–1.05)	(0.99–1.04)	(0.98–1.04)	(0.78–1.31)	(0.75–1.27)	(1.12–1.52)	(1.02–1.51)
**Venezuela**	574	1.01	1.01	1.03	1.03	1.11	1.08	1.17	1.16
		(0.97–1.05)	(0.98–1.04)	(1.00–1.06)	(0.99–1.05)	(0.93–1.33)	(0.91–1.30)	(1.03–1.34)	(1.00–1.35)
**Meta-effect**	4590	1.02	1.02	1.02	1.01	1.11	1.11	1.18	1.14
		(1.01–1.03)	(1.01–1.03)	(1.00–1.04)	(1.00–1.03)	(0.96–1.25)	(0.95–1.26)	(1.07–1.29)	(1.02–1.26)
**I^2^**		14.0%	20.8%	71.2%	44.7%	70.2%	70.7%	64.9%	64.4%

**Notes:** “China (rural)” was excluded because there were too few cases to estimate parameters; References in comparisons were a one point increase on the EURO-D or a 10-point increase on the WHODAS-II, where appropriate; **^1^** Includes contacts with primary care, private doctor, hospital doctor, traditional healer, other services, in the previous 3 months; **^2^** Because the total score is expressed as a percentage, WHODAS-II scores were adjusted to 10-point prevalence; **^3^** Adjusted for age (groups), gender, education, and physical comorbidities; MV = Number of missing values.

**Table 4:** Association of individual functioning difficulties (WHODAS-II items) with any community health service use **^1^**.

No.	Item	Pooled (Meta-Analysed) Prevalence Ratios (95% CI)					
		Crude		I^2^ (%)	Adjusted^2^		I^2^ (%)
		PR	(95% CI)		PR	(95% CI)	
1	Difficulty with standing	1.02	(0.99–1.04)	0	1.01	(0.99–1.03)	0
2	Difficulty with taking care of household responsibilities	0.99	(0.96–1.01)	0	0.98	(0.96–1.01)	0
3	Difficulty with learning a new task	0.99	(0.96–1.01)	0	0.99	(0.97–1.02)	0
4	Difficulty with joining in community activities	0.99	(0.95–1.04)	60.2	1.00	(0.96–1.04)	55.2
5	Emotionally affected by health problems	1.06	(1.03–1.09)	31.7	1.04	(1.01–1.07)	22.4
6	Difficulty with concentrating on doing something for ten minutes	1.00	(0.98–1.03)	7.3	1.01	(0.98–1.03)	0
7	Difficulty with walking a long distance	1.01	(0.97–1.04)	60.4	1.00	(0.97–1.04)	51.5
8	Difficulty with washing whole body	0.93	(0.89–0.98)	28.9	0.93	(0.88–0.98)	37.6
9	Difficulty with getting dressed	1.01	(0.97–1.05)	1.7	1.01	(0.97–1.05)	0
10	Difficulty with unknown people	0.93	(0.85–1.01)	73.3	0.93	(0.86–1.01)	71.0
11	Difficulty with maintaining a friendship	1.02	(0.96–1.07)	54.4	1.01	(0.95–1.06)	49.2
12	Difficulty with day-to-day work/school	1.03	(0.99–1.06)	25.7	1.03	(0.99–1.06)	34.7

**Notes:** References in comparisons were a one point increase on individual WHODAS-II items; **^1^** Includes contacts with primary care, private doctor, hospital doctor, traditional healer, other services, in the previous 3 months; **^2^** Adjusted for age (groups), gender, education, and physical comorbidities; PR = Prevalence ratios.

To account for the potential impact of depressive symptoms on yielded mutually-adjusted associations of specific functioning difficulties with “any community HSU”, sensitivity analyses were conducted. Here WHODAS-II items relating to depressive symptoms (*i.e.*, item 5: emotionality; item 6: concentration) were removed from the adjusted models. These analyses found no additional significant associations of specific functioning difficulties with increased “any community HSU”. However, “difficulty with washing whole body” remained significantly associated with decreased “any community HSU” (Pooled PR = 0.93; 95% CI = 0.89–0.98; I^2^ = 37.0%)

## 4. Discussion and Conclusions

### 4.1. Summary of Findings

After adjustment for confounding variables, depressive symptom severity was significantly associated with “any community HSU” but not hospital admission. After adjustment, functioning was significantly associated with hospital admission but not “any community HSU”. When investigating individual functioning difficulties, only “emotionally affected by health problems” was significantly associated with increased “any community HSU”, whereas “difficulty with washing whole body” was associated with decreased “any community HSU”.

### 4.2. Limitations and Strengths

Some methodological limitations should be considered when interpreting the findings. The cross-sectional nature of the study meant it was not possible to determine if depressive symptom severity and functioning predict HSU over time. Relatedly, depression symptom severity and functioning data pertained to a retrospective one-month period whereas HSU data pertained to a retrospective three-month period. Therefore, yielded associations did not take into account potential fluctuations in depression symptom severity and functioning over the entire three-month investigative period. The WHODAS-II was used to measure functioning but due to the wide-ranging operationalisation of functioning in the literature, it is possible that other functioning measures (e.g., “activities of daily living”) would have yielded markedly differing associations with HSU if used in the present study. Because hospital-based services are analogous to community-based services in various study sites, the outcome of “any community HSU” included services not typically set in the community in high-income countries: the findings relating to the hospital admissions outcome are more applicable to high income countries than those relating to the “any community HSU” outcome. Due to an insufficient number of available cases to estimate parameters, it was not possible to determine the association of functioning items from the WHODAS-II with hospital admission. Finally, as reported using Higgins I^2^ [35], heterogeneity at site level varied in analyses of study outcomes and for some outcomes it was at a high level (Table 3 and Table 4), perhaps reflecting differences in access to care. This heterogeneity limits somewhat the validity of findings from conducted meta-analyses. However, regression models adjusted for variables associated with access to care in LMICs such as education and pension coverage [23], and similar access-related variables such as “number of assets” and private insurance were reported for each site.

The study benefits from its large sample size, representative of populations of older people living in urban and rural catchment areas in nine LMICs, with minimal missing data. The inclusion of only those participants in the clinical range for depressive symptoms adds precision to yielded associations, as compared with previous studies using general population samples. Moreover, the study is one of few investigations of the association of mental health and HSU, in LMICs. Therefore, the findings could be influential in a region in which momentum for health care reforms aimed at achieving universal health coverage is growing [24]. Finally, sensitivity analyses of the association of depressive symptom severity (using “previous ICD-10 depressive episode”) with HSU yielded similar results to analyses of this association involving the EURO-D [26]. This adds validity to results concerning (EURO-D) depressive symptom severity and HSU.

### 4.3. Comparison of Findings with Previous Research

The mixed evidence for an association of depressive symptom severity with HSU by older people with depression reflects the inconsistent evidence from previous research [11]. The significant association of depressive symptom severity with “any community HSU” is in line with some community-based general population studies [10,14] but not others [13]. The absence of a similar association with hospital admissions contradicts results from a study involving older men with elevated depressive symptoms living in Australia [12] but corroborates results from a study involving older people registered with a health maintenance organisation in USA [14]. The significant association of depressive symptom severity with “any community HSU” yielded a small effect size (PR = 1.02), similar to the findings of a previous meta-analysis of the association of these symptoms with hospital admissions which also yielded a small effect size (RR = 1.36) [11]. Overall, the findings suggest that depressive symptom severity does not explain a large amount of the variance in HSU by older people with depression, which highlights the need to investigate the influence of other variables on this HSU.

In relation to functioning, no previous research on the association of functioning with HSU by older people with depression was available for direct comparison of results. The absence of an association of functioning with “any community HSU” contradicts general population studies which found associations of physical impairments with community-based HSU by older people living in LMICs [23], and “activities of daily living” with community-based HSU by adults of varying age in the general population [17]. These contrasting findings may be partly explained by the differences in the operationalisation of functioning across these studies. The finding of a significant association of functioning with hospital admissions in the present study accords with previous results showing that functioning (operationalised as “activities of daily living”) is associated with hospital-based service use by medical inpatients [17]. However, the validity of this comparison is limited due to differing sample compositions and differing operationalisations of functioning. Overall, results from the present study and those from previous research provide preliminary evidence for an association of functioning with HSU by older people with depression, suggesting this association is worthy of further investigation in future research.

The finding that only one functioning item from the WHODAS-II was significantly associated with increased “any community HSU” is partly explained by the absence of an association of the WHODAS-II total score with “any community HSU”. More associations relating to individual WHODAS-II functioning items would likely have been found if it was possible to undertake analysis using the outcome of hospital admission, because the WHODAS-II total score was significantly associated with this outcome. Nevertheless, the functioning item significantly associated with increased HSU concerned emotionality, and this is in line with the earlier finding from this study and previous research showing an association of depressive symptom severity with increased HSU [11]. The finding that “difficulty with washing whole body” was associated with decreased “any community HSU” was unexpected, but this difficulty implies a lack of independence which could represent a barrier to accessing community-based health services. No previous research directly investigating an association of individual functioning items/ difficulties with HSU by older people with depression was available for further comparison of results.

### 4.4. Implications of Findings for Practice

The findings, added to those from previous research, have implications for health service planning. As these findings suggest that depressive symptom severity is not strongly related to HSU by older people with depression, the formation of relevant patient clusters (*i.e.*, groupings of patients with similar clinical characteristics and HSU patterns used in contemporary health service payment systems to allocate resources) should take into account additional patient-related variables. Moreover, health services should consider functioning to be a possible determinant of HSU by older people with depression, and functioning information should be investigated further to assess its suitability for patient clustering. The overall modest associations of depressive symptom severity and functioning with HSU suggest that health service planning for patients with depression is potentially a complex process. Only a few high income countries (e.g., Australia, New Zealand, Canada, the Netherlands, Norway, USA, UK) have made progress implementing mental health payment systems, but with widely varying methodologies [36]. Therefore, it is important that more studies investigate the potential broad range of patient-related variables that may be related to HSU by older people with depression.

The relatively modest associations also point to the complexity of estimating HSU by specific cohorts in LMICs with no universal health care coverage. In these settings, several sociodemographic variables relating to health service accessibility (e.g., private health insurance coverage, pension coverage, education) are associated with HSU by older people [23] and these variables may explain substantially more of the variance in HSU than either symptom severity or functioning.

### 4.5. Future Research

Attempts to replicate the study using longitudinal data should make it possible to determine if depressive symptom severity and functioning predict HSU by older people with depression in LMICs over time. Based on our results and (the little) available evidence from longitudinal studies in various populations [11], it is expected that positive associations of depressive symptom severity and functioning with HSU would be found, although the magnitude of these associations may be small. Similar studies involving participants from younger age groups and high income countries would increase the applicability of findings to diverse health services. Because differing operationalisations of functioning may account for equivocal findings across studies, the association of differing functioning measures (as well as individual items) with HSU by older people with depression is worth investigating. The functioning measure—PARADISE-24 [37] developed to account for the hypothesis of “horizontal epidemiology”, namely that common functioning difficulties are typically experienced across all neurological and mental disorders, may be suitable for this endeavour. Findings relating to a study investigating the association of the PARADISE-24 with HSU would be applicable to a wide range of mental disorders [37]. Taking into account the range of integrated services needed to effectively treat mental health problems, future research investigating the link between mental health and HSU could include a wider range of HSU outcomes than used in the present study.
